# Identification and expression analysis of *YABBY* family genes in *Platycodon grandiflorus*

**DOI:** 10.1080/15592324.2022.2163069

**Published:** 2023-01-22

**Authors:** Lingyang Kong, Jiaying Sun, Zhehui Jiang, Weichao Ren, Zhen Wang, Meiqi Zhang, Xiubo Liu, Lijuan Wang, Wei Ma, Jiao Xu

**Affiliations:** a Pharmacy of College, Heilongjiang University of Chinese Medicine, Harbin, China; b School of Forestry，Northeast Forestry University, Harbin China; c College of Jiamusi, Heilongjiang University of Traditional Chinese Medicine (TCM), Jiamusi, China; d Ophthalmology Hospital in Heilongjiang province, Harbin, China; e Key Laboratory of Basic and Application Research of Beiyao (Heilongjiang University of Chinese Medicine), Ministry of Education, Harbin, China

**Keywords:** YABBY transcription factor, *Platycodon grandiflorus*, abiotic stress, qRT-PCR, expression patterns

## Abstract

*Platycodon grandiflorus* set ornamental, edible, and medicinal plant with broad prospects for further application development. However, there are no reports on the YABBY transcription factor in *P. grandiflorus*. Identification and analysis of the *YABBY* gene family of *P. grandiflorus* using bioinformatics means. Six *YABBY* genes were identified and divided into five subgroups. Transcriptome data and qRT-PCR were used to analyze the expression patterns of *YABBY. YABBY* genes exhibited organ-specific patterns in expression in *P grandiflorus*. Upon salt stress and drought induction, *P. grandiflorus* presented different morphological and physiological changes with some dynamic changes. Under salt treatment, the *YABBY* gene family was down-regulated; *PgYABBY5* was up-regulated in leaves at 24 h. In drought treatment, *PgYABBY1, PgYABBY2*, and *PgYABBY3* were down-regulated to varying degrees, but *PgYABBY3* was significantly up-regulated in the roots. *PgYABBY5* was up-regulated gradually after being down-regulated. *PgYABBY5* was significantly up-regulated in stem and leaf at 48 h. *PgYABBY6* was down-regulated at first and then significantly up-regulated. The dynamic changes of salt stress and drought stress can be regarded as the responses of plants to resist damage. During the whole process of salt and drought stress treatment, the protein content of each tissue part of *P grandiflorus* changed continuously. At the same time, we found that the promoter region of the *PgYABBY* gene contains stress-resistant elements, and the regulatory role of YABBY transcription factor in the anti-stress mechanism of *P grandiflorus* remains to be studied. *PgYABBY1, PgYABBY2*, and *PgYABBY5* may be involved in the regulation of saponins in *P. grandiflorus. PgYABBY5* may be involved in the drought resistance mechanism in *P. grandiflorus* stems and leaves. This study may provide a theoretical basis for studying the regulation of terpenoids by the YABBY transcription factor and its resistance to abiotic stress.

## Introduction

*Platycodon grandiflorus* is a perennial herb of the *Platycodon* genus in the Platycodon family. It is mainly distributed in Northeast China, North Korea, Japan, Russia, etc., and is used as an auxiliary ingredient in dishes or used directly in pickles. At present, more than 100 kinds of compounds, such as steroidal saponins, flavonoids, phenolic acids, polyacetylenes, and sterols, have been isolated from *P. grandiflorus*. Triterpenoid saponins are the main active components in *P. grandiflorus*, including platycodins D, E, A, etc. It is one of the main triterpenoids^1^. which have many pharmacological benefits, such as enhancing immune stimulation, anti-inflammation, anti-obesity, anti-atherosclerosis, and anti-cancer effects. In addition, *P. grandiflorus* has been used to treat many chronic diseases, such as bronchitis, asthma, and tuberculosis.^2^ *P. grandiflorus* polysaccharides (PGPs) are another important active ingredient in this species, which are related to the antioxidant activity; for instance, selenium polysaccharides in *P. grandiflorus* may be a potentially useful antioxidant.^3,4^ In addition, PGPs can activate macrophages and enhance nonspecific immune functions.^5^

YABBY is a family of unique transcription factors in plants, which has two highly conserved domains in its members: the zinc finger-like domain and the YABBY domain.^6–8^ The zinc finger domain is located at the N-terminal of the YABBY protein, and the YABBY domain is located at the C-terminal. The zinc finger structure belongs to the C2C2 type, and there are gaps of 20 amino acids between cysteine pairs. The YABBY domain has a helix-loop-helix structure,^4,9^ which constitutes the first two of the three α helices in the high mobility group (HMG) box. Based on the different expression patterns of this gene in angiosperms, it can be divided into two types: the vegetative and reproductive types.^10,11^ The ‘vegetative’ genes regulate the polar development of lateral organs, formation of edges, the growth of meristem and leaves at the stem tips, and the maturation of leaves.^12–14^ The reproductive type includes *CRABS CLAW* (*CRC*) and *INNER-NO-OUTER* (*INO*), which are only expressed in developing carpels and ovules, respectively.^15,16^ According to the phylogenetic analysis of YABBY proteins in different species, the six YABBY members in *Arabidopsis thaliana: FILAMENTOUS FLOWER* (*FIL), YABBY3* (*YAB3), CRABS CLAW* (*CRC), INNER NO OUTER* (*INO), YABBY2* (*YAB2*), and*YABBY5*.^17–19^ In a study on *A.thaliana, FIL, YABBY2, YABBY3*, and *YABBY5* were observed to be expressed in both vegetative and reproductive organs.^20^

The *YABBY* gene has been established to play an important role in the development and growth of plants. An *FIL, OsYABBY4*, is mainly expressed in rice vascular tissues to regulate vascular development,^21^ while *YABBY2* member in *Oryza sativa, OsYABBY1, FIL/YAB3* members in *Zea mays, ZYABBY9* and *ZYABBY14*, and *YABBY2, YABBY3*, and *YABBY5* in *A. thaliana* have redundant functions to promote the development of collateral organs.^13,22^ *CRC* is restricted to the developing carpel and nectaries in *A. thaliana*.^15^ *ZmYABBY1* and *ZmYABBY11* regulate the development of male florets,^23^ while drooping leaves in rice regulate the development of carpel and the formation of the midvein.^24^ *CRC* members in rice and corn also have conservative functions in leaf development, whereby they affect leaf width, length, angle, and internode diameters.^25,26^ *INO* promotes the development of ovule ectoderm into the seed coat.^15^ In *A. thaliana, INO* participates in the formation and development of the exocarp.^27^

*YABBY* genes are also involved in plant hormone responses. For example, overexpression of *OsYABBY1* in rice leads to a semi-dwarf phenotype through the feedback regulation of gibberellin (GA), as well as its biosynthesis and metabolism;^28^ *OsYABBY4* regulates plant development and growth by regulating GA signaling pathways.^21^ *YABBY* genes are also involved in abiotic stress. For example, over-expression of *AcYABBY4* from pineapple in *A. thaliana* can negatively regulate the salt tolerance of plants.^29^ Genome-wide analysis of the *YABBY* gene in kidney beans showed that they were involved in salt stress;^30^ similarly, *GmYABBY10* in soybean was noted to negatively regulate drought and salt tolerance in plants.^31^ For example, molecular cloning results presented by Takahiro Yamaguchi and other researchers have shown that *DL* is a member of the *YABBY* gene family that is closely related to *CRC* in *A. thaliana* based on evolutionary relationships. During the process of rice flower development, carpel development is regulated by *DL*. When the function of *DL* is seriously deficient, a complete homeotic transformation^28^ from carpel to stamen occurs, without affecting the characteristics of other flowering organs.

This study presents findings from bioinformatics analysis of the *YABBY* gene family in *P. grandiflorus*. The dynamic changes in the differences in *PgYABBY* expression were analyzed under salt and drought stress, laying a foundation for the study of YABBY transcription factors that may be involved in the regulation of terpenoids and in imparting resistance against abiotic stress in *P. grandiflorus*. Our results will provide theoretical support for follow-up studies on secondary metabolites of medicinal plants to optimize the breeding of *P. grandiflorus* varieties.

## Materials and methods

### Materials

The whole genome data of *P grandiflorus* (GCA_016624345.1) comes from NCBI (https://www.ncbi.nlm.nih.gov/) database. It is acquired from the 4th generation inbred of Jangbaek-doraji. The YABBY protein sequence information for *A. thaliana* (GCA_000005425.2) was obtained from the Plant TFDB (http://planttfdb.cbi.pku.edu.cn/) database, while the transcriptome data of the different tissue parts were obtained by self-test.

### *Identification and analysis of* PgYABBY

Repeated transcripts caused by variable shearing were removed to obtain the member ID of the *YABBY* gene family. The protein sequences of the *YABBY* family proteins from *P. grandiflorus* were then obtained by TB tools^32^ software. The size, relative molecular weight, theoretical isoelectric point, and hydrophilic average coefficient of the YABBY protein sequence of *P. grandiflorus* were predicted on the ExPASy website (https://web.expasy.org/protparam/). WoLFPSORT website (http://www.genscript.com/wolf-psort.html) was used to predict the subcellular localization of *YABBY* family members. (**Supplementary Table 1**). Homology modeling techniques are widely used in protein models. To determine the tertiary structure of YABBY protein, we used the fully automated protein structurehomology modeling server Phyre2 database (http://www.sbg.bio.ic.ac.uk/phyre2) for^33^ homology modeling.

### Multiple sequence comparison and phylogenetic analysis of PgYABBY Protein

Multiple sequence alignment of all identified YABBYs in *P. grandiflorus* was carried out using ClustalW,^34^ and a phylogenetic tree was generated by neighbor-joining (NJ) method with default parameters: bootstrap method setting to 1000, Poisson model, and complete deletion in MEGA 11.The conserved domain of a YABBY protein, PgYABBY, in *P. grandiflorus* was compared and analyzed by using DNAMAN software. The data of this YABBY protein for *A. thaliana* were obtained from the Plant TFDB database, which was then merged with the file of the YABBY protein of *P. grandiflorus*. Multi-sequence alignment was carried out by the MUSCLE program. The neighbor-joining (NJ) method was performed using MEGA11^35^ software with a Bootstrap value of 1000 repetitions to construct a phylogenetic tree.

### *Analysis of* PgYABBY *gene structure and conserved motif*

TB tools were used to analyze *YABBY* gene structure, and online software, MEME (http://meme-suite.org/tools/meme), was used to predict the motif, where the maximum motif number was set to 10. After setting other default parameters, TB tools was used to visualize the genetic structure and motifs in the evolutionary tree.

### *Prediction of* PgYABBY Cis*-acting elements*

TB tools was used to extract the 2000-bp sequence upstream of the target *YABBY* gene in *P. grandiflorus*, considering it the promoter region. (**Supplementary Table 2**) The sequence was submitted to Plant Care (http://bioinformatics.psb.ugent.be/webtools/plantcare/html/) as predicted *cis*-acting elements. the result files were screened, classified, and visualized.

### *Analysis of* PgYABBY *gene expression*

The transcriptome data of leaves, petals, pistils, roots, seeds, sepals, stamens, and stems of Landrace Doraji were standardized, followed by cluster analysis. The heat map was drawn using TB tools.

### cDNA preparation

Total Plant RNA Kit (product serial number 5101050) was purchased from Hangzhou Xinjing Biochemical Reagent Development Co., Ltd. (Hangzhou China) The first strand cDNA High-Efficiency Synthesis (Reverse Transcription) Kit (article number QP057) was purchased from Guangzhou Yijin Biotechnology Co., Ltd. (Guangzhou China). RNA was extracted according to the methods and steps described by Hangzhou Xinjing Biochemical Reagent Development Co., Ltd. After extracting RNA, it was stored at −80°C. For analysis, a proper amount of RNA was the reverse transcription reagent was melted while being kept on ice. The reverse transcription program was applied as follows: 25°C for 5 min, 42°C for 15 min, 85°C for 5 min, and 4°C for the holding step. The concentration of cDNA products was measured by an ultra-micro ultraviolet spectrophotometer, and cDNA was stored in the refrigerator at −20°C for later use.

### Real-time quantitative experiment

BlazeTaqTMSYBR®GreenqPCRMix2.0 Real-Time Fluorescence Quantitative PCR Detection Reagent (article number QP031) was purchased from Guangzhou Yijin Biotechnology Co., Ltd. According to the laboratory instructions of the reagent company.In order to study the expression patterns of *PgYABBY* genes under stress, a real-time quantitative fluorescence test was performed on the annually bred P. *grandiflorus* that had been subjected to different treatments. cDNA was diluted according to the experimental requirements, and real-time quantitative PCR was performed using the manufacturer’s instructions. PCR on each sample was repeated three times. PrimerPrimer5 software was used to design primers, and the Tm value was kept between 55°C and 60°C. PCR was performed to verify whether the primers were effective. Primers used in the *P. grandiflorus* expression pattern experiment were synthesized by Harbin Qingke Jiamei Biotechnology Co., Ltd. (Harbin China) (**Supplementary Table 3**).

## Results

### *Basic information on YABBY Genes in* P. grandiflorus

**Figure 1.**
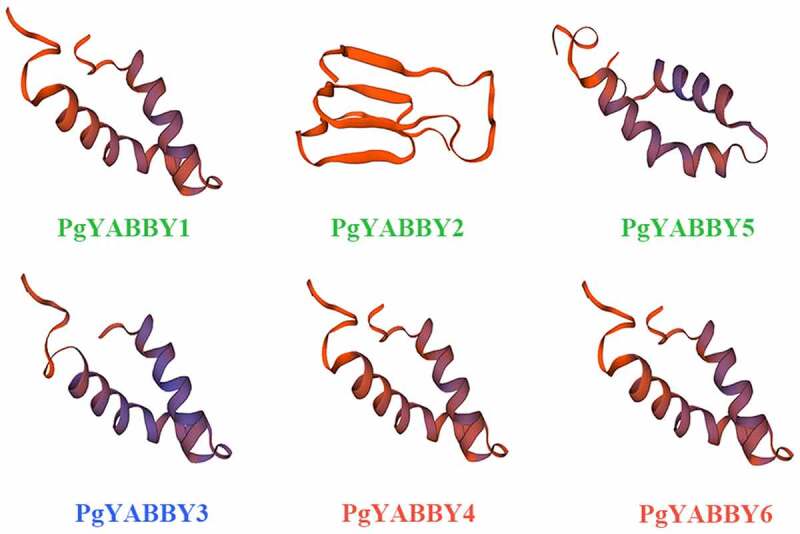
Tertiary structure analysis of YABBY proteins.

Each *YABBY* gene in *P. grandiflorus* was given a transcript accession number. Using basic information on the *YABBY* gene family in *P. grandiflorus*, it was found that their sizes are between 183 and 277 aa. The proteins encoded by *PgYABBY* genes are predicted to be located in the nucleus, their isoelectric points were between 4.72 and 9.38, while their relative molecular weights were different. YABBY proteins are hydrophilic with different hydrophilicities, and the aliphatic amino acid index was observed to be between 61.86 and 77.42.(Table 1) The tertiary structure analysis of *P. grandiflorus YABBY* gene shows that the members of the subfamily are similar in structure, and the proteins containing the helix domain bound to CCDC124-80S-eERF1 ribosome complex are highly similar. There are also differences in the structure of different subfamily genes, For example, different proteins have different structures (Figure 1)

**Table 1.** Information and characteristics of *PgYABBY* genes.

Gene-name	Locus-name	aa	Location	pI	M.W (D)	GRAVY
PgYABBY1	PGJG176780.1	213	nucleus	8.61	23660.89	−0.381
PgYABBY2	PGJG176790.1	225	nucleus	5.72	23991.7	−0.13
PgYABBY3	PGJG200940.1	219	nucleus	7.19	24261.59	−0.412
PgYABBY4	PGJG306470.1	277	nucleus	5.31	31052.56	−0.733
PgYABBY5	PGJG327920.1	183	nucleus	9.38	20640.26	−0.694
PgYABBY6	PGJG350850.1	213	nucleus	5.64	24206.19	−0.599

The diagram shows the tertiary structure of six YABBY proteins from three subfamilies.

### Phylogenetic analysis and classification

**Figure 2..**
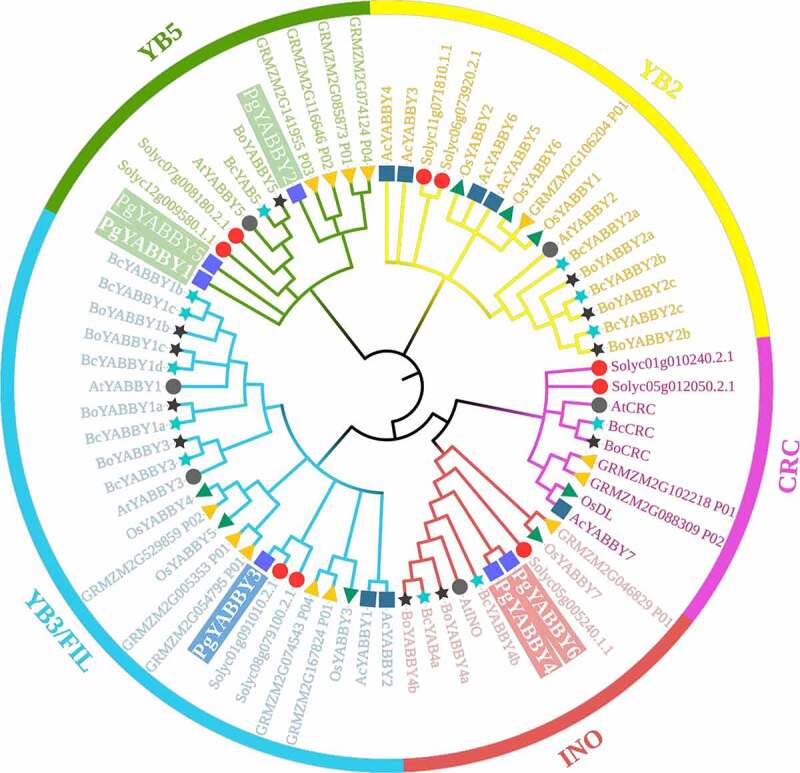
Phylogenetic tree of interspecific YABBY relationships.

To better explore the evolutionary relationship of *YABBYs*, six *Arabidopsis YABBYs*,eight rice *YABBYs*, nine *Solanum lycopersicum YABBYs*,thirteen *Zea mays YABBYs*, eleven *Brassica oleracea YABBYs*, twelve *Pak-choi YABBYs*, seven *Ananas comosus YABBYs*, and six *YABBY* members in *P. grandiflorus* were used to construct an evolutionary tree using MEGA 11 with ClustalW and NJ methods. As reported in Arabidopsis, the *P. grandiflorus YABBYs* were also divided into five subfamilies, *YAB1, YAB2, CRC, INO*, and *YAB5* (Figure 2). The *FIL* and *YAB2* subfamilieshad the larger numbers of *YABBYs*, in which the *FIL* subfamily contained 24 members while *YAB2* subfamily contained 17 members. Subfamilies of *CRC* shared the smallest *YABBYs* with nine members, respectively. *P. grandiflorus YAB1, YAB2* and *YAB5* are all divided into *YAB5* subfamily, *YAB4* and *YAB6* are all distributed in *INO* subfamily, and finally only *YAB3* belongs to *FIL* subfamily (Figure 1 **and** Table 1). Taken together, these results suggested that there are evolutionary splits and diversifications of *YABBYs* among different species.

In MEGA 11, phylogenetic trees without roots were developed using the neighbor-joining (NJ) process. At each node, bootstrap values of 1,000 replicates are indicated. Dark gray triangle represents the YABBY sequence of *A.thaliana*, green right pointing triangle represents the YABBY sequence of *Oryza sativa*, red circle represents the YABBY sequence of *Solanum lycopersicum*, yellow left pointing triangle represents the YABBY sequence of *Zea mays*, black star represents the YABBY sequence of *Brassica oleracea*, green star represents the YABBY sequence of *Pak-choi*, blue square represents the YABBY sequence of *Ananas comosus*, and finally purple square represents the YABBY sequence of *P. grandiflorus*. (Figure 2)

### Relationship between PgYABBY Protein sequence alignment and evolution

**Figure 3.**
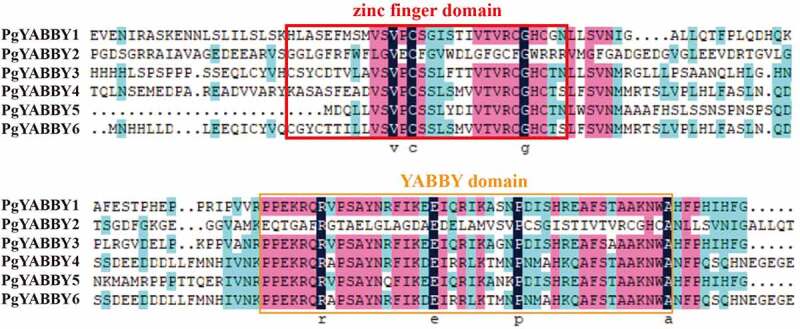
Sequence of the *P. grandiflorus* YABBY proteins. Two conserved regions were identified, including the C2C2 zinc finger region at the N-terminal and the YABBY domain at the C-terminal. The zinc finger-like domain.

PgYABBY1, PgYABBY3, PgYABBY4, PgYABBY5, and PgYABBY6 all have complete conservative domains, and PgYABBY2 contains only some conservative amino acids, such as valine, cysteine, and glycine in the zinc finger domain, arginine, glutamic acid, proline, and aspartic acid in the YABBY domain (Figure 3).

### Conserved motifs and gene structure in the PgYABBY protein

**Figure 4.**
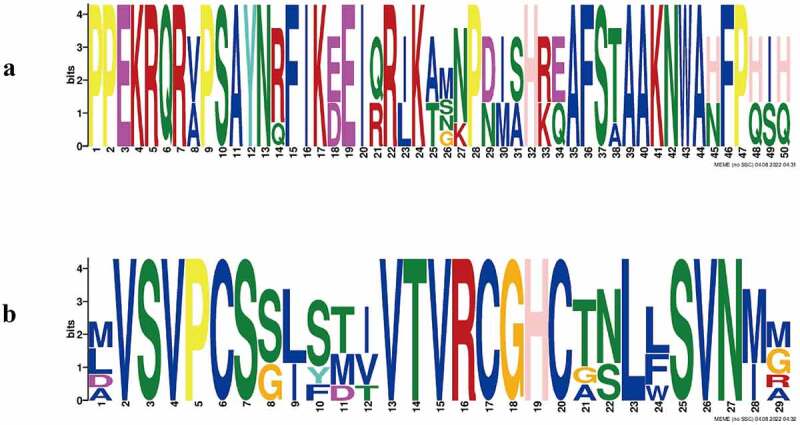
**a** conserved motifs in motif1 protein. **b** the conservative motifs in Motif2 protein.

A total of 10 conserved motifs were found in the YABBY proteins of *P. grandiflorus* (**Supplementary Figure 1**), among which Motif1 (Figure 4a) and Motif2 (Figure 4b) were highly conserved. Each protein contained Motif2, and all proteins, except PgYABBY2, contained Motif1. The conserved amino acids of Motif1 include proline, glutamic acid, lysine, arginine, serine, alanine, and phenylalanine, while those of Motif2 include valine, serine, proline, threonine, cysteine, glycine, histidine, and leucine.

**Figure 5.**
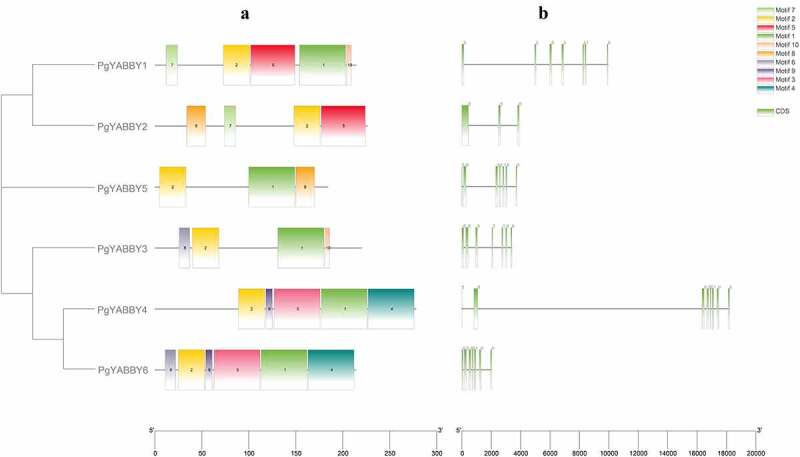
Conserved motifs in PgYABBY proteins and the gene names on the left are arranged in order and divided by green color background. CDS, coding sequence. Introns are represented by black lines.

According to the evolutionary relationships among PgYABBY proteins (Figure 5), it can be inferred that closely related proteins have similarly conserved motifs (Figure 5a), For instance, PgYABBY4 and PgYABBY6, which belong to the *INO* subfamily contain Motif1, Motif2, Motif3, and Motif9, where the motifs are arranged in the same order. PgYABBY1 and PgYABBY2 both contain Motif2, Motif5, and Motif7, while PgYABBY1 and PgYABBY5 both contain Motif1 and Motif2. PgYABBY2 and PgYABBY5 both contain Motif8. PgYABBY3 contains three conserved motifs, including Motif6, in addition to the two highly conserved motifs.

Visualizations of the structure of the *YABBY* genes in *P. grandiflorus* using TB Tools software showed that *PgYABBY2* has two very special introns, while other *YABBY* gene structures were conserved. The number of introns was generally 6–7, of which only *PgYABBY4* contained seven introns. The introns were extremely long, and the rest of the members contained only six introns. (Figure 5b)

### *Prediction of* Cis*-elements of the* PgYABBY *gene*

**Figure 6.**
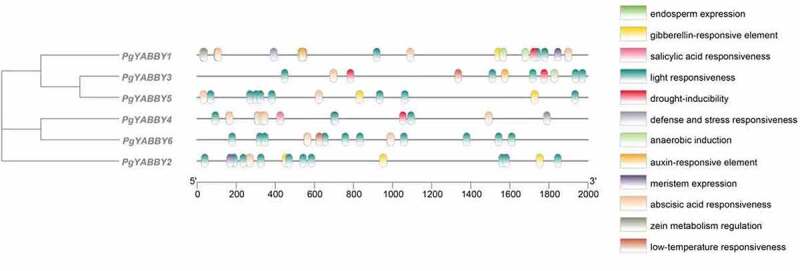
The *cis*-acting elements within the promoters of *PgYABBYs.*

The promoter region of the *YABBY* genes in *P. grandiflorus* contained a variety of *cis*-elements (Figure 6). Each *YABBY* gene contained light-responsive elements, which constitute the largest number of elements, followed by some hormonal *cis*-elements. *PgYABBY1* contained the greatest number of different kinds of cis-elements, including 11 kinds. These specifically include elements dealing with defense and stress response, zein metabolism regulation, and endosperm expression. *PgYABBY2* contained five cis-acting elements, most of which are photo-response elements. The *PgYABBY3* promoter region contained six elements. *PgYABBY4* had four *cis*-acting elements, where the number of abscisic acid-responsive elements was second only to that of photo-response elements. *PgYABBY5* and *PgYABBY6* contained only three *cis*-acting elements, both of which contained light-responsive elements and abscisic acid-responsive elements.

### *Expression Pattern of the* PgYABBY *gene*

**Figure 7.**
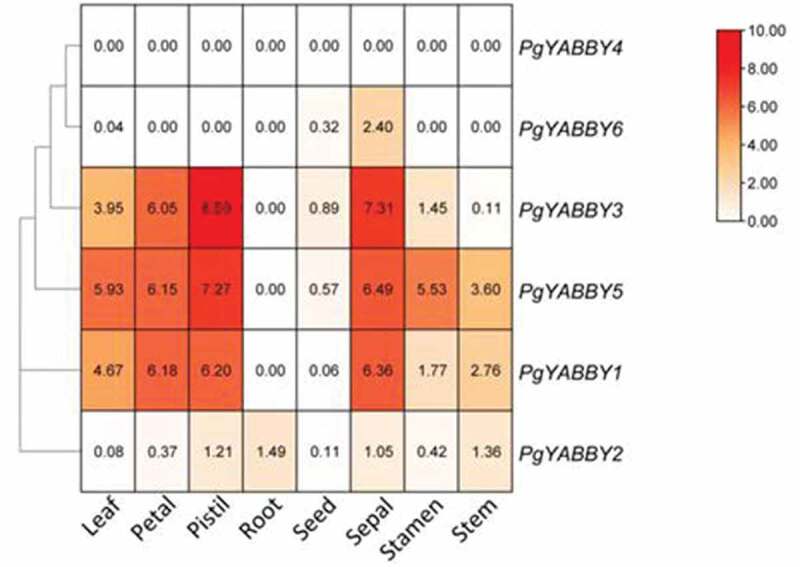
Thermograph of the expression of *YABBY* genes in the different Organs of *P. grandiflorus*. Root; Stem; Seed; Stamen; Sepal; Pistil; Leaf; Petal.

From the heat map (Figure 7), we can infer that the expression of *PgYABBY1* in sepals, leaves, pistils, and petals was higher than that in other tissue parts. The FPKM values in petals, pistils, and sepals were all higher than 6.0. *PgYABBY1, PgYABBY2*, and *PgYABBY5* are homologous genes. *PgYABBY2* was only slightly expressed in various tissue parts, and the highest expression level was recorded in the roots. *PgYABBY5* was very similar to *PgYABBY1* in its expression pattern, but it was only weakly expressed in roots and seeds. The expression of *PgYABBY3* in pistil was the highest with an FPKM value of 8.50, followed by sepals, where the FPKM value was 7.31. *PgYABBY4* was not expressed in all tissues. The expression of *PgYABBY6* in sepals was ~8 times higher than that in seeds, but there was no expression in other tissues.

### *Expression patterns of the* YABBY *gene under salt and drought stress*

**Figure 8.**
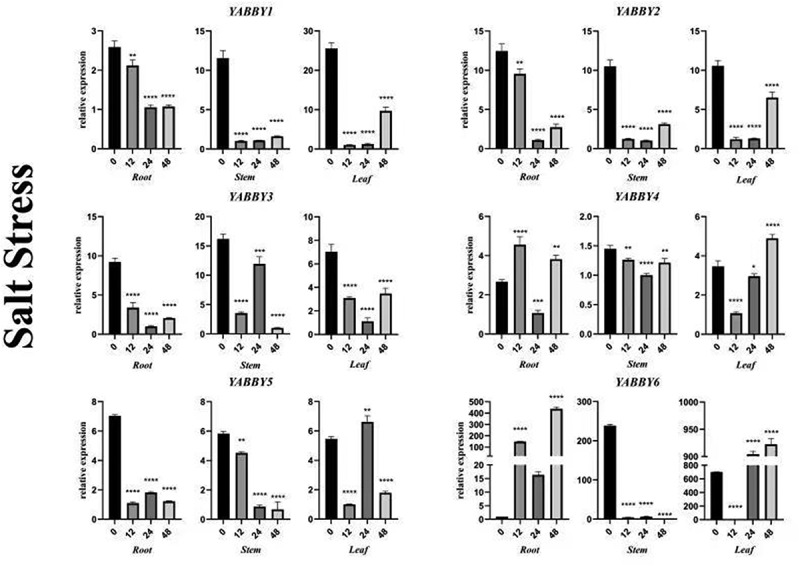
qRT-PCR analysis of *YABBY* genes under salt stress at different time points. The horizontal coordinates indicate the different time points and the vertical coordinates indicate the relative expression levels. t-test demonstrated that statistically significant differences: *p < .05, **p < .01, ***p < .001, ****p < .0001.

Under salt stress, the expression of the *PgYABBY1* gene in the root showed a downward trend (Figure 8), and it became stable after 24 hours of treatment. The expression level of the *PgYABBY1* gene in the stem was significantly down-regulated at 12 h. The expression levels of the *PgYABBY1* gene remained low with extension in treatment time. The expression of this gene in leaves was significantly down-regulated by about 25 times at 12 h and showed an upward trend after 48 h of treatment.

The changing trend of *PgYABBY2* in the three tissue parts under salt stress was basically the same, and the expression level was down-regulated as a whole. At the root, *PgYABBY2* was down-regulated within 24 h and was up-regulated at 48 h after treatment. In the stem, *PgYABBY2* decreased significantly after 12 h of treatment and then increased slightly. In the middle leaves, *PgYABBY2* decreased significantly at 12 and 24 h and increased at 48 h. Salt stress also inhibited the expression of *PgYABBY3* in *P. grandiflorus*. In the root, *PgYABBY3* was down-regulated and slightly up-regulated. In the stem, *PgYABBY3* showed an upward trend at 24 h after being strongly down-regulated, and then was down-regulated finally. *PgYABBY3* was down-regulated in the leaves at first and then increased after 48 h.

At the root, *PgYABBY4* increased at first followed by a significant decrease at 24 h, and then increased significantly at 48 h. Salt stress had little effect on the expression of *PgYABBY4* in stems, and its expression reached the lowest level after 24 h of salt stress. In the leaves, *PgYABBY4* decreased to the lowest level at 12 h and then increased continuously. Under the influence of salt stress, root *PgYABBY5* was down-regulated, although it had an upward trend in 24 h; however, the amplitude remained small. *PgYABBY5* was down-regulated in the middle stem. *PgYABBY5* showed a significant downward trend at 12 h in leaves, then increased significantly, and finally decreased sharply at 48 h. Salt stress has a strong influence on the expression of *PgYABBY6*. After 12 h of treatment, the expression of *PgYABBY6* in root was significantly up-regulated, then down-regulated at 24 h, and then finally up-regulated. In the stem, the expression of *PgYABBY6* was down-regulated; it slightly increased at 24 h and then decreased to the lowest level at 48 h. In the middle leaves, *PgYABBY6* decreased significantly at 12 h, then increased significantly, and remained stable till the 48th hour. After salt stress treatment, the expression pattern of the *YABBY* genes in different tissue parts was analyzed. It was found that the expression of the *YABBY* genes in the three different tissues, root, stem, and leaf, changed with a change in stress treatment time, indicating that the *YABBY* gene can play different roles and functions in coping with abiotic stress in *P. grandiflorus*.

### *Analysis of expression pattern of* YABBY *gene under drought stress*

Drought stress can obviously inhibit the expression of *PgYABBY1*. The expression of *PgYABBY1* in roots, stems and leaves was down-regulated, especially in stems, which was down-regulated by about 650 times at 48 h.

**Figure 9.**
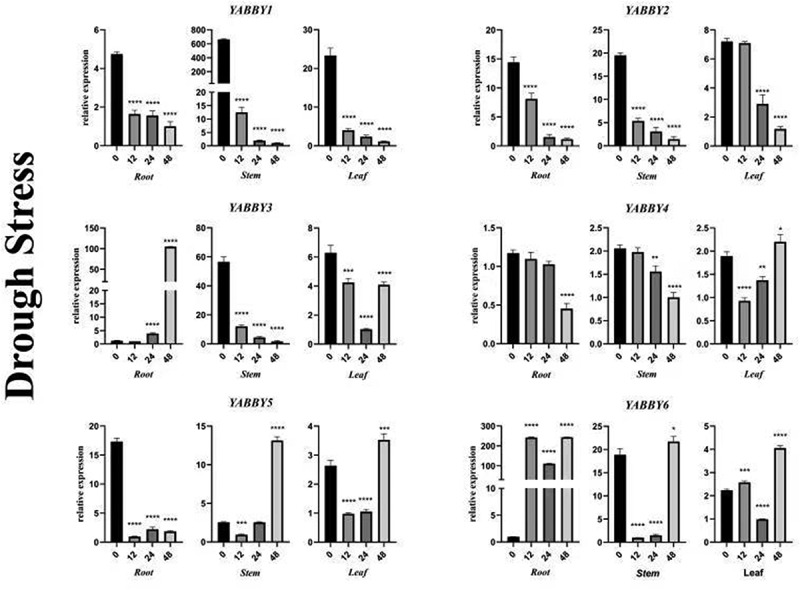
qRT-PCR analysis of *YABBY* genes under drought stress at different time points. The horizontal coordinates indicate the different time points and the vertical coordinates indicate the relative expression levels. t-test demonstrated that statistically significant differences: *p < .05, **p < .01, ***p < .001, ****p < .0001.

The expression of *PgYABBY2* was also inhibited by drought stress; it always showed a downward trend in roots, stems, and leaves, especially when the leaves were treated for 24 h. In the roots, with an increase in exposure time to drought stress, *PgYABBY3* generally showed an upward trend, and it significantly increased at 48 h. Drought stress inhibited the expression of *PgYABBY3* in stems, where its expression levels decreased continuously. In leaves, *PgYABBY3* was down-regulated first and then up-regulated. During the whole treatment process, the expression of *PgYABBY4* was not obvious; its expression level in roots and stems was continuously down-regulated. In leaves, *PgYABBY4* was down-regulated at 12 h and then up-regulated. Drought stress inhibited the expression of *PgYABBY5* in roots; its expression showed a downward trend during treatment. *PgYABBY5* was slightly expressed in the stem at first and was up-regulated after 48 h of exposure to drought. The expression level of *PgYABBY5* in middle leaves was low from 12 to 24 h, and it was up-regulated by about 3 times at 48 h. The *PgYABBY6* gene was significantly up-regulated in roots with change in treatment time; it was up-regulated by nearly 250 times at 12 h, down-regulated at 24 h, and then increased again. *PgYABBY6* was significantly down-regulated at 12 h in the stem, then up-regulated continuously. It was up-regulated ~20 times at 48 h. In leaves, *PgYABBY6* was first up-regulated and then down-regulated, and then up-regulated by ~4 times. Therefore, different observations were made on the *YABBY* genes in different tissue parts under drought stress. It was found that the expression of the *YABBY* genes in the three different tissue parts, root, stem, and leaf, can change with the change in exposure time (Figure 9), indicating that the *YABBY* genes can play different roles in coping with drought stress in *P. grandiflorus*.

## Discussion

In this study, a total of six *YABBY* genes were identified in *P. grandiflorus*. Each *P. grandiflorus* gene was assigned a transcript accession number, and the number of amino acids encoded by them was between 183 and 277, which is similar to the number of amino acids encoded by the YABBY genes reported in other species, such as *Arabidopsis*.^29^ Except for *PgYABBY2*, which had only a few conserved amino acids, all *P. grandiflorus* YABBY proteins featured YABBY and zinc finger domains.^36^ Two highly conserved motifs were found in the YABBY proteins, which were speculated to be related to the conserved domains of the YABBY transcription factor and the YABBY and zinc finger domains. *PgYABBY2* contained only one conserved motif, rendering it different from other PgYABBY proteins, but the reason for this difference remains unclear. The number of introns in the *PgYABBY* genes was relatively conserved, and only the *PgYABBY2* gene presented a special structure. Other *PgYABBY* genes were consistent with the reported *YABBY* genes in different species,^36^ indicating that the *YABBY* genes are conserved in different species.

The upstream promoter region of *P. grandiflorus* contained a variety of *cis*-acting elements, among which light- and abscisic acid-responsive elements were distributed in each *P. grandiflorus YABBY* gene. The light-responsive elements were more numerous, indicating that the *YABBY* genes may be affected by light during the growth and development of *P. grandiflorus. PgYABBY1* contained the most abundant *cis*-acting elements, such as some hormones and development-related elements. In addition, it specifically contains three elements: those related to defense, stress response, zein metabolism regulation, and endosperm expression, which indicates that *PgYABBY1* may be related to stress resistance and developmental regulation in plants. The elements related to meristem expression exist only in *PgYABBY1* and *PgYABBY2*, indicating that these two genes may be involved in inducing meristem expression. which suggesting that these genes might be involved in different developmental processes, which was consistent with the study by Dai et al.^37^ Genes containing drought-inducing elements may be involved in plants’resistance to drought stress.

*YABBY* genes can act as pivotal regulatory factors involved in the leaf evolution in plants. They regulate various developmental processes, such as restriction of meristem apex, polarity, layered development, the establishment of leaf margin, flower differentiation, carpel formation, outer carpel growth, inflorescence, etc.^8,38^ The YABBY transcription factor gene plays an indispensable regulatory role in the formation of lateral organs of plant, while boosting their development.^39^ It was found that the YABBY family members play a key role in the development of leaves and leaf-derived organs, such as cotyledons and flowers.^40^

Gene structure is always conserved during the process of evolution.^41,42^ Our results showed that the density of splicing of the regulatory sequences increased with the increase in intron length (<1.5 kb), and the increase in intron length (>1.5 kb) was related to the increase in splicing site strength.^43^ The intron patterns across the different genes may play an evolutionary role in expression conservation or splicing modulation in *P. grandiflorus*. Furthermore, the intron burden of evolutionarily conserved genes has been reported to be large, and the degree of evolutionarily conserved eukaryotic genes is positively correlated with the size of the intron region.^44^ Similarly, the range of expression of *FIL/YABBY3*-like, *YABBY2*-like, and *YABBY5*-like genes in Myrica rubra was wider than that of *CRC*-like and *INO*-like genes, which may take part in more significant biological functions, which is consistent with previous studies on *A. thaliana*.^13^

*Cis*-elements in the promoter region play an important role in gene expression regulation. The presence or absence of these elements affects gene expression.^45^ The conserved *cis*-acting motif can be used to forecast the function and underlying interaction of genes.^46^ The conserved cis-acting motif can be used to forecast the function and underlying interaction of genes.^10,13^ In order to better comprehend the regulation of *ACYABBY*, the *cis*-elements of its promoter region were studied. Many types of regulatory elements were found in the putative promoter of *ACYABBY*, including elements related to stress response, hormone, reproduction, circadian rhythm control, and other regulatory mechanisms. MeJA and ABA regulate the growth and development of plants, while regulating the defense of plants against trauma, disease, infiltration, and other adverse environmental factors.^47,48^

The expression levels of *ACYABBYS* have been reported to be distinct from that of other plants. For instance, *CRC* and *INO* are only in the reproductive development stage of *Ataliaana, Bienertia rapa*, and *B. sinuspersici*, disclosing their conservative functions in carpel morphogenesis, pistil polarization, florals meristem, and ectodermal development.^17,49^ In *P. granatum, PgCRC* is strongly expressed in leaves, androgynous flowers, functional stamens, and skin, especially in leaves and androgynous flowers, while the flower-specific gene, *PgINO*, is strongly expressed only in androgynous flowers and exocarp; it is only weakly expressed in roots and exocarp.^17^ In addition, previous studies have shown that *FIL, YABBY2*, and *YABBY3* are related to the distal domain of leaf-derived organs, such as cotyledons, leaves, and flowers.^8,12^ The *FIL/YABBY3*-like *YABBY* gene has been proven to be involved in the maintenance of meristem function.^50,51^ *OsYABBY4* is defined as the branch of *FIL/YABBY3*, which may take part in the vascular system of rice because of its dominant position in the phloem.^52^ The homologous genes, *AcYABBY5* and *AcYABBY6*, related to *FIL* in carambola have no specific expression regions.^53^ In tomatoes, the *FAS* gene is similar to the *YABBY2* and is crucial to the size and shape of the fruit.^54^ Similar to *FAS*, the expression of the *YABBY2*-like gene in reproductive organs of carambola was noted to be higher than that in the vegetative organs. *YABBY1* is similar to *YABBY5*, which is highly expressed in the flowers and fruits of carambola during diverse progressive stages; it may be involved in the development of carambola fruit. The main active ingredients in *P. grandiflorus* are terpenoids, namely the *P. grandiflorus* saponin, which is an important active ingredient in *P. grandiflorus* and has high medicinal value. The secondary metabolism^55^ of plants plays a very important role in plant growth and development, adaptation to external environmental changes, biological interactions, and stress responses. Secondary metabolites are not only the result of plants adapting and evolving to suit their environment, but are also essential components of most medicinal plants. Glands and glandular hair^55^ are important organs for plant secondary metabolite production; they can synthesize, secrete, and store secondary metabolites. Transcription factors^53^ can regulate the secondary metabolism of plants.

According to previous research, YABBY transcription factors have dual functions and can act as activators and repressors of secondary metabolites. Terpenoids are multifunctional compounds in plant secondary metabolites. Similarly, phenolic compounds are also a kind of representative secondary metabolites with unique physiological functions. Anthocyanins are the most common flavonoids amongst the phenolic compounds. In *A. thaliana, FIL* can positively regulate anthocyanins,^56^ especially the accumulation of anthocyanins induced by JAZ protein in tissues, depending on the activity of the *YABBY* gene. In spearmint, *MsYABBY5* can reverse regulate terpenes in plants.^55^ *AaYABBY5* in *Artemisia annua* has a positive regulatory effect on artemisinin (sesquiterpene lactones).^57^ The regulation of terpenoids by *YABBY* genes has not been studied, and the production of secondary metabolites by plants is also related to abiotic and biotic stress factors. The abiotic stress treatment of *P. grandiflorus* was carried out to analyze the expression pattern of the *YABBY* genes in response to salt and drought stress. This provided a molecular basis for further study on the regulation of terpenoid secondary metabolites by YABBY transcription factors from the perspective of increasing the content of medicinally active components in *P. grandiflorus* by genetic engineering. Such investigation will also help achieve the goal of reducing research and development and the production costs of drugs related to terpenoid-based active components. The *MsYABBY5*^55^ and *AaYABBY5* of the *YABBY5* subfamily have negative and positive regulatory effects on terpenoids, respectively, therefore we speculate that the *PgYABBY* of the *YABBY5* subfamily plays a regulatory role in Platycodon species.

Salt stress inhibited the expression of *PgYABBY6*, while it showed a positive response in roots and leaves, indicating that *PgYABBY6* was probably involved in the regulation mechanism of *P. grandiflorus* against salt stress. Drought only inhibited the expression of *PgYABBY1* and *PgYABBY2*; with the extension of drought treatment time, *PgYABBY3* in roots increased significantly, indicating that *PgYABBY3* could respond positively to severe drought stress in roots. It could be used as a candidate gene in studying the drought resistance mechanism of *P. grandiflorus* roots. After 48 h of treatment, *PgYABBY5* was up-regulated in the stems and leaves, proving that proper drought stress could increase the expression of this gene. *PgYABBY6* responded positively to both salt and drought stress, which is of great significance to further explore the abiotic stress resistance mechanism of *P. grandiflorus.*

## Conclusion

In this study, six *YABBY* genes were identified in *P. grandiflorus*, which is in line with the characteristics of small gene families. The characteristics of *YABBY* genes are conservative, and the number of introns is roughly (6 or 7). All YABBY proteins contain conservative motifs, and the number of amino acids is between 180 and 300 aa. There are many elements related to hormones and stress upstream of *CsYABBY* and *PgYABBY* at 2000 bp, indicating the expression of YABBY transcription factors in different plants. Through the genome-wide identification and analysis of the YABBY transcription factor in *P. grandiflorus*, the influence and function of the YABBY transcription factor on the growth and development of *P. grandiflorus* plant organs have been predicted. It is speculated that these genes are involved in the regulation of terpene secondary metabolites, which is helpful to further studying the function of *YABBY* genes. Based on salt and drought stress, the expression analysis of the *YABBY* gene in *P. grandiflorus* can lay a foundation for further exploring the mechanism of the *YABBY* gene response to abiotic stress.

## Supplementary Material

Supplemental Material

## Data Availability

All data generated or analyzed during this study are included in this published article and its supplementary information files https://doi.org/10.1080/15592324.2022.2163069.
