# Supplementary material for: Identification and expression analysis of YABBY family genes in Platycodon grandiflorus
Source: Plant Signal Behav. 2023 Jan 22;18(1):2163069. doi: 10.1080/15592324.2022.2163069 (PMC9870009; doi:10.1080/15592324.2022.2163069)
Supplement: Supplemental Material [file KPSB_A_2163069_SM6877.zip › Supplementary Table 3.doc]

**Supplementary Material**

**__**

| number | SEQUENCE | LENGTH |
| --- | --- | --- |
| PgYAB1-F | ACCTTCTTTCGGTCAACATG | 20 |
| PgYAB1-R | CTGCATTTAGACGACGACGA | 20 |
| PgYAB2-F | GGTTTGGGGTTTCTTTAGGC | 20 |
| PgYAB2-R | GCTTACCATGGCCAACTCAT | 20 |
| PgYAB3-F | GCCCATTTCCCTCACATCCA | 20 |
| PgYAB3-R | AAACACCCAAGTTGGCTGGA | 20 |
| PgYAB4-F | CTCCCTGCTCGCTAAGAATG | 20 |
| PgYAB4-R | ACATCAGCCTCAAAGGATGC | 20 |
| PgYAB5-F | ATGGACCAGCTTTTGGTGAG | 20 |
| PgYAB5-R | TGGTTAGGACCACCCTGAAG | 20 |
| PgYAB6-F | AGCCTTCAGTACAGCTGCAA | 20 |
| PgYAB6-R | GCCTTCTGCATGGACCTCTT | 20 |
| GADPH-F | CAGGGAGGCTTTTAGTTCAGGT | 20 |
| GADPH-R | ATCACATCTACACCCCTCCAGC | 20 |
