# Supplementary figures and images for: Identification and expression analysis of YABBY family genes in Platycodon grandiflorus
Source: Plant Signal Behav. 2023 Jan 22;18(1):2163069. doi: 10.1080/15592324.2022.2163069 (PMC9870009; doi:10.1080/15592324.2022.2163069)

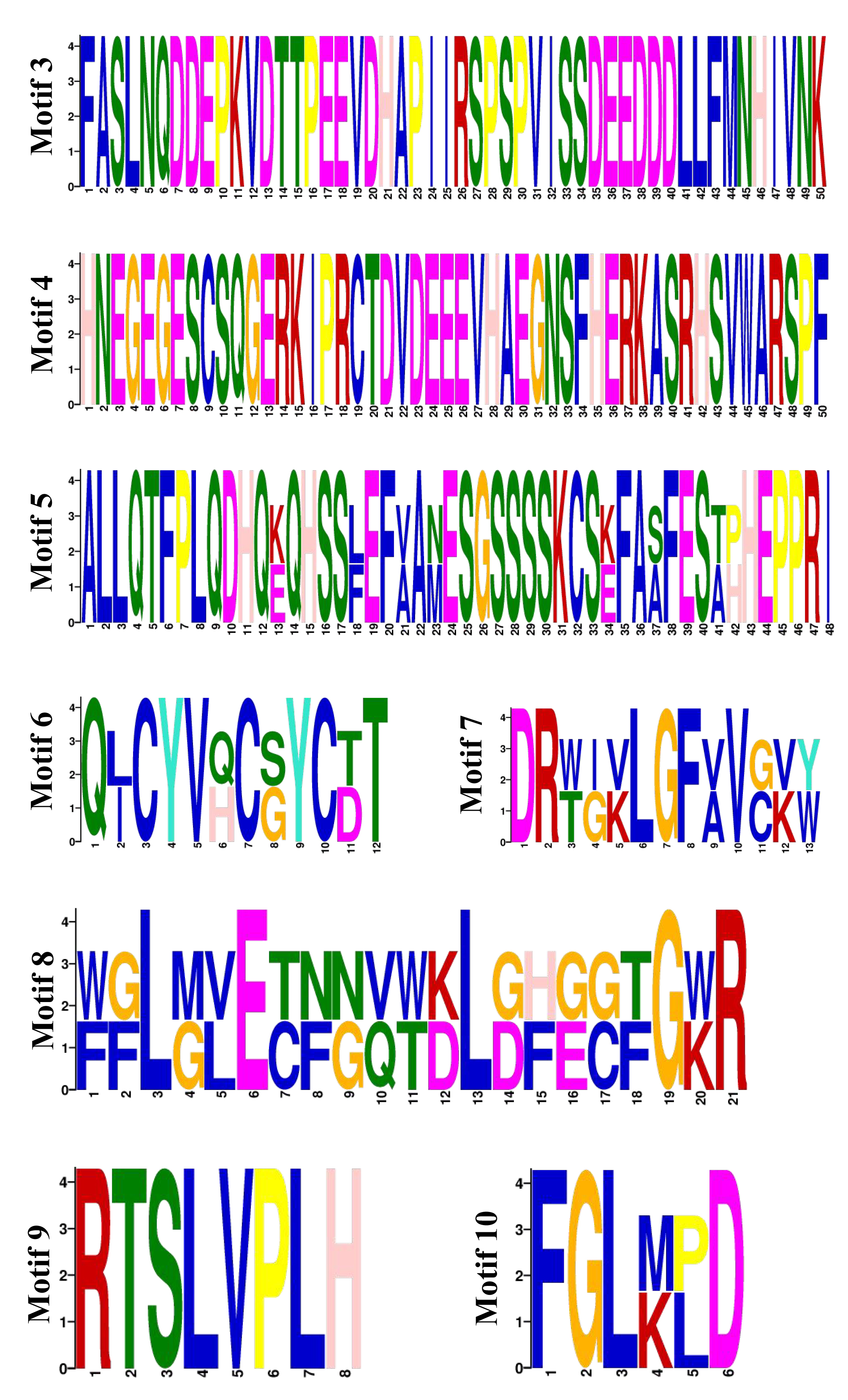

Supplement: Supplemental Material [file KPSB_A_2163069_SM6877.zip › Supplementary Fig 1.jpg]
